# Aerosolized Intratracheal Inoculation of Recombinant Protective Antigen (rPA) Vaccine Provides Protection Against Inhalational Anthrax in B10.D2-Hc^0^ Mice

**DOI:** 10.3389/fimmu.2022.819089

**Published:** 2022-01-26

**Authors:** Xiaolin Song, Wei Zhang, Lina Zhai, Jianshu Guo, Yue Zhao, Lili Zhang, Lingfei Hu, Xiaolu Xiong, Dongsheng Zhou, Meng Lv, Wenhui Yang

**Affiliations:** State Key Laboratory of Pathogen and Biosecurity, Beijing Institute of Microbiology and Epidemiology, Beijing, China

**Keywords:** anthrax, vaccine, mouse model, dry powder formulation, aerosolized intratracheal inoculation

## Abstract

Anthrax caused by *Bacillus anthracis* is a fatal zoonotic disease with a high lethality and poor prognosis. Inhalational anthrax is the most severe of the three forms of anthrax. The currently licensed commercial human anthrax vaccines require a complex immunization procedure for efficacy and have side effects that limit its use in emergent situations. Thus, development of a better anthrax vaccine is necessary. In this study, we evaluate the potency and efficacy of aerosolized intratracheal (i.t.) inoculation with recombinant protective antigen (rPA) subunit vaccines against aerosolized *B. anthracis* Pasteur II spores (an attenuated strain) challenge in a B10.D2-Hc^0^ mouse (deficient in complement component C5) model. Immunization of rPA in liquid, powder or powder reconstituted formulations *via* i.t. route conferred 100% protection against a 20× LD_50_ aerosolized Pasteur II spore challenge in mice, compared with only 50% of subcutaneous (s.c.) injection with liquid rPA. Consistently, i.t. inoculation of rPA vaccines induced a higher lethal toxin (LeTx) neutralizing antibody titer, a stronger lung mucosal immune response and a greater cellular immune response than s.c. injection. Our results demonstrate that immunization with rPA dry powder vaccine *via* i.t. route may provide a stable and effective strategy to improve currently available anthrax vaccines and B10.D2-Hc^0^ mice challenged with *B. anthracis* attenuated strains might be an alternative model for anthrax vaccine candidate screening.

## Introduction

*Bacillus anthracis*, a gram-positive, non-motile, facultative aerobic bacteria, is the causative agent of anthrax (1). It is also of great concern as a biological weapon and categorized by the Centers for Disease Control (CDC) as a category A biological threat agent (2). Anthrax is a fatal zoonotic disease primarily observed in ungulates and humans (3). Depending on the route of exposure, there are three forms of anthrax: cutaneous, gastrointestinal and pulmonary (4–6). Pulmonary anthrax, also called inhalational anthrax, is the most severe of the three forms with mortality rates for untreated human cases approaching 100%, compared with 10-20% for the cutaneous form. After being inhaled and deposited within the alveolar spaces of the host respiratory tract, spores of *B. anthracis* are taken up by macrophages and dendritic cells, and transported to lymph nodes, where they germinate into vegetative cells, followed by bacillar multiplication, dissemination and toxin production (7). The release of toxins leads to anthrax, which manifests as sepsis, septic shock or meningitis.

The currently licensed United Kingdom and United States human anthrax vaccines are prepared from the cell-free culture supernatant of attenuated *B. anthracis* strains V770-NP1-R and Sterne 34 F2, followed by adsorption to aluminium hydroxide gel or precipitate of potassium aluminium sulphate. To develop and maintain protective immunity in humans, these vaccines call for a series of six doses within 18 months *via* s.c. injection and require yearly boosters (8, 9). They are also associated with local side effects and provide partial protection against infection with some strains of *B. anthracis* in animal models (10, 11). The development of a more effective, easily administered, and safer vaccine would thus be of great benefit, especially given the malicious release of anthrax spores in the 2001 terrorist attack in the US (12–14).

*B. anthracis* virulence is due to two major components, the poly-gamma-D-glutamic acid capsule and the tripartite anthrax toxin, comprised of protective antigen (PA), lethal factor (LF), and edema factor (EF) (15). PA plays a central role in the formation of lethal toxin (PA+LF) and edema toxin (PA+EF). Without PA, the toxin cannot be translocated into the host cell cytosol to exert its cytotoxic effect. Therefore, development of a second-generation anthrax vaccine is focused on a subunit vaccine of recombinant PA (rPA) (16, 17). Although the subunit vaccine of PA gives good protection in both rabbit and non-human primate models, the best vaccine composition and administration procedure needs to be further studied (15, 18–20). Different formulations, various adjuvants and delivery systems are among some of the strategies being explored (19, 21).

Administration of rPA *via* intramuscular (i.m.) injection or s.c. injection induces low levels of antibody. Recently, increasing attention has been focused on pulmonary delivery of vaccines due to their ability to recruit the local immune responses of the bronchopulmonary mucosa in addition to the systemic immune response (22). For this delivery method, liquid formulations of vaccines require cold-chain for storage and transport to maintain vaccine potency, while powder formulation offers the potential to eliminate preservatives and the cold-chain requirement, maintaining long term stability for room temperature storage and shipping (23–27).

To evaluate the immunogenicity and protective efficacy of anthrax vaccine, a suitable animal model is required. An ideal experimental animal model uses a specific host species with increased sensitivity to a defined strain. A number of animal models have been used for evaluation of protection against anthrax infection, including mice (28, 29), guinea pigs, rabbits (30, 31) and Rhesus macaques (32), most of which required use of biosafety level 3 (BSL-3) or higher laboratories because of the high virulence of this bacterium. Unfortunately, only a few laboratories are equipped with the requirements for this level of biosafety, limiting the advances of such research. Studies have shown that different mice strains exhibit different susceptibility to anthrax infection. Mice lacking a functional Hc gene, which encodes for complement component C5, are sensitive to anthrax infection by an attenuated *B. anthracis* strain, the Sterne strain (28, 33). Complement depletion also makes C57BL/6 mice sensitive to the Sterne strain (34). Thus, B10.D2-Hc^0^ H2^d^ H2-T18c/oSnJ mice (hereafter referred to as B10.D2-Hc^0^), which are deficient in complement component C5, were selected as a potential model for the initial screening of our vaccines.

In this study, we prepared rPA with the adjuvant CpG oligodeoxynucleotide (CpG) into a spray-freeze-dried (SFD) powder formulation suitable for aerosolized i.t. inoculation (35, 36). We then assessed the efficacy of different rPA formulations (liquid, powder and reconstituted powder) for immunization *via* different immunization routes (i.t. and s.c.) against an attenuated *B. anthracis* Pasteur II strain spore challenge in the B10.D2-Hc^0^ mice model. The results provide insight on formulations and delivery routes that deserve further consideration as an improved anthrax vaccine and demonstrate a useful small animal model for anthrax vaccine candidate screening.

## Materials and Methods

### Animals

Pathogen-free, female B10.D2-Hc^0^ mice (6 to 8 weeks old) were obtained from Jackson Laboratory and maintained in our laboratory. All procedures involving animals were conducted in accordance with and approval from the Beijing Institute of Microbiology and Epidemiology, and the ethical approval number was IACUC-IME-2021-030. Before the experiments, the mice were acclimatized to the laboratory conditions for 1 week.

### Preparation of rPA Dry Powder

Formulations containing rPA and CpG were prepared as SFD powder using previously described methods (37). Briefly, rPA and CpG were dissolved in an aqueous solution of the excipients containing D-mannitol, myo-inositol, L-leucine, and poloxamer 188 at a 1:1 wt/wt ratio. The pH value was adjusted to 7.2 with NaOH solution (1 mol/L). The solution was kept in an ice bath for 2 h and then passed through a two-fluid pneumatic spray nozzle (2 mm diameter, TSE Inc, Thuringia, Germany) at a liquid feed rate of 5 mL/min. At a height of 10 cm below the nozzle, a circular stainless vessel containing liquid nitrogen collected the droplets. The sprayed atomized droplets were quickly frozen into ice crystals under the action of liquid nitrogen. The ice crystals, together with a small amount of remaining liquid nitrogen, were transferred to a stainless-steel cup for lyophilization in a vacuum freeze-drying system for 48 h. The dry-powder formulations were stored at 4°C until use.

The rPA dry powder was reconstituted in deionized water and analyzed by SDS-PAGE and Western blot (using polyclonal antibodies collected from rPA-immunized mice). Particle morphology was observed under multiple fields of view with a scanning electron microscope. The volume median diameter (VMD) of dry powder vaccines was determined by a laser particle size analyzer (RODOS&HELOS, Sampytec, Germany) and the mass median aerodynamic diameter (MMAD) of rPA aerosol particles was measured by the aerodynamic particle sizer (APS) spectrometer 3321 (TSI Inc, Minnesota, USA). The moisture content in the rPA dry powder sample was determined by thermogravimetric analysis (TGA).

### Immunization Procedures

**Figure 1:**
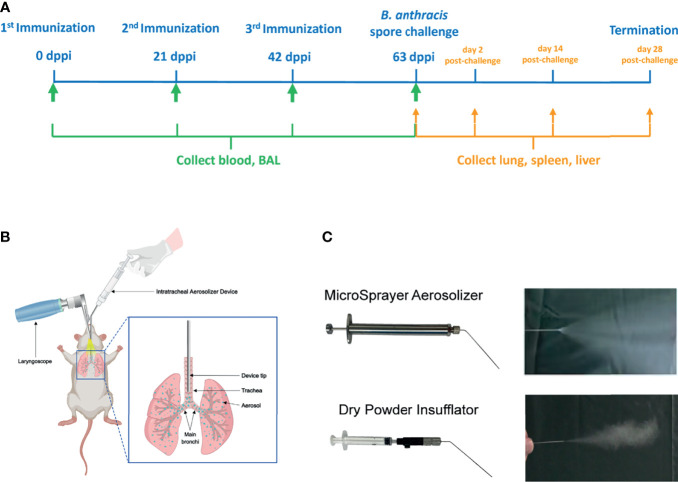
Schematic representation of the immunization protocol. **(A)** The immunization scheme. Mice were immunized three times at 3-week intervals and challenged with aerosolized *B. anthracis* Pasteur II spores 3 weeks after the third immunization. Serum and BAL were collected before each immunization and before the *B*. *anthracis* spore challenge for antibody analysis. Lung, spleen and liver were collected at 21 days after the third immunization (63 dppi) and on days 2, 14, and 28 post-challenge for histopathology and spore load analysis. **(B)** Schematic of aerosolized intratracheal inoculation. **(C)** Two Intratracheal Aerosolizer Devices and their effects of generating aerosols in the air. BAL, bronchoalveolar lavage; dppi, days post-primary immunization.

B10.D2-Hc^0^ mice were immunized *via* i.t. or s.c. on days 0, 21 and 42 of the experiment (**Figure 1A**). Mice were assigned to one of ten groups (five experimental groups, three negative controls and two blank controls) with 44 mice in each group (see **Table 1**). The five experimental groups included three groups of mice inoculated using i.t. delivery with either 1) 0.5 mg of rPA dry powder (i.t.-rPA, powder), 2) 0.5 mg of rPA dry powder reconstituted in PBS (i.t.-rPA, powder reconstituted), or 3) 20 μg of rPA liquid in PBS (i.t.-rPA, liquid). The remaining two groups of mice were inoculated using s.c. delivery with either 4) 0.5 mg rPA dry powder reconstituted in PBS (s.c.-rPA, powder reconstituted), or 5) 20 μg of rPA liquid in PBS (s.c.-rPA, liquid). Mice in negative controls were immunized with CpG and those in blank controls were immunized with PBS. In our powder vaccine, rPA as well as CpG contributed to 4.0% of the total mass (See **Table S1** in the electronic **Supplementary Material** for details), indicating that 0.5 mg of powder contains 20 μg of rPA.

**Table 1:** Summary of all immunization groups used in the experiment.

Immunization route	Group	Formulation type	rPA dose (μg/mouse)	CpG dose (μg/mouse)	Volume (μL/mouse)
i.t.*^a^*	rPA	Powder	20	20	50
i.t.	rPA	Powder reconstituted	20	20	50
i.t.	rPA	Liquid	20	20	50
i.t.	CpG	Powder	/	20	50
i.t.	CpG	Powder reconstituted	/	20	50
i.t.	CpG	Liquid	/	20	50
i.t.	PBS	Liquid	/		50
s.c.*^b^*	rPA	Liquid	20	20	100
s.c.	rPA	Powder reconstituted	20	20	100
s.c.	CpG	Liquid	/	20	100
s.c.	CpG	Powder reconstituted	/	20	100
s.c.	PBS	Liquid	/		100

^a^i.t., aerosolized intratracheal inoculation.

^b^s.c., subcutaneous injection.

For i.t. immunization, mice were anesthetized by intraperitoneal injection of pentobarbital sodium (70 mg/kg of body weight). Then each anesthetized mouse was placed on the slanted board in supine position. When the tracheal opening was clearly viewed by a laryngoscope (Huironghe Company, Beijing, China), the Intratracheal Aerosolizer Device (Huironghe Company, Beijing, China) was inserted 25 mm from the larynx (near the tracheal bifurcation) for vaccine delivery, followed by the uniform dispersion of vaccine throughout the lung (**Figure 1B**). There are two kinds of Intratracheal Aerosolizer Devices, MicroSprayer Aerosolizer and Dry Powder Insufflator, suitable for liquid formulation and powder formulation, respectively (**Figure 1C**). They could eject the drug as an aerosol with the dose of 50 μL suspension or a certain mass of powder for one administration. For s.c. injection, each mouse was subcutaneously injected with 100 μL of vaccine suspension into the inner thigh.

### Determination of Anti-rPA IgG and SIgA

Serums and bronchoalveolar lavage fluids (BAL) from four mice per group were collected before each immunization and before the *B. anthracis* spore challenge. The titers of rPA-specific IgG and SIgA antibodies in serum and mucosal samples were measured by enzyme-linked immunosorbent assay (ELISA) as described previously (37).They were calculated as the reciprocal of the lowest sample dilution equal to 2.1 times the background optical density (OD) values. Background values were obtained using samples collected from naive mice.

### Toxin-Neutralizing Antibody (TNA) Assay

TNA titers were estimated using of a modified version of a method described elsewhere (38). J774A.1 cells were plated (5×10^3^ cells/well) in sterile, 96-well, clear-bottom plates (Corning Costar) at 37°C in 5% CO_2_. A fresh solution containing 5 μg/mL LF (List Biological Laboratories) and 10 μg/mL rPA was mixed with an equal volume of diluted samples in duplicate and incubated for 1 h at 37°C. Then, 10 μL of above mixed sample was added to each well, and wells were incubated for 4 h at 37°C in 5% CO_2_. Cell viability was assessed with a CCK8 assay. End-point percent neutralization was calculated using the formula: (sample OD value – LeTx standard OD value)/(cells-only OD value - LeTx standard OD value) × 100. The OD of a medium-only well was subtracted from all values before percent neutralization was calculated. A 4-parametric sigmoid regression curve was used to determine the dilution of antiserum that resulted in a 50% reduction in toxicity of anthrax LeTx.

### Cytokine Level Measurement

Three mice per group were euthanized at 63 days post-primary immunization (dppi). Total mononuclear cells were isolated from spleens and suspended (1×10^6^/mL) in DMEM basic medium (Gibco, Shanghai, China) containing 10% (v/v) fetal bovine serum (Gibco, Australia) and penicillin-streptomycin (Gibco, Grand Island, USA) in a 96-well ELISPOT plate (Mabtech, Nacka Strand, Sweden). Then, 10 μg/mL rPA, 2.5 μg/mL Concanavalin A (ConA, positive control, Sigma-Aldrich, Darmstadt, Germany) or cell culture medium (negative control) was added to wells, which were next incubated for 18 h at 37°C under 5% CO_2_. All measurements were performed in at least triplicate. interferon-γ (IFN-γ) and interleukin-4 (IL-4) levels were measured by enzyme-linked immunospot (ELISPOT) assays as described elsewhere (37).

### Spore Preparation

*B. anthracis* strain Pasteur II was cultured in Luria–Bertani (LB) medium. Overnight cultures were inoculated with LB at 1:40 and cultured at 26°C for 6 days. The culture was heat-treated at 65°C for 40 min to kill any viable vegetative cells. Spores were then washed extensively in distilled water to remove inactive vegetative cells and spores were stored at -20°C for subsequent quantification and use.

### *B. anthracis* Aerosol Challenge

For the anthrax infection experiment, B10.D2-Hc^0^ mice were randomly divided into five groups (10 mice per group) that were infected with different doses of *B. anthracis* Pasteur II spores using i.t. delivery. The operation of i.t. delivery of the spores was the same as i.t. immunization. Observations continued for 14 days, and deaths recorded daily for LD_50_ calculation.

In the vaccine effectiveness evaluation experiment, at 63 dppi, *B. anthracis* (Pasteur II strain) spores were enumerated and diluted for aerosolized challenge. Mice immunized with rPA, CpG or PBS were challenged intratracheally with 5×10^4^ CFU (20× LD_50_) or 1×10^5^ CFU (40× LD_50_) *B. anthracis* spores in 50 uL of PBS. Animals were closely monitored for signs of weakness and survival for 14 days.

At days 2, 14 and 28 post-challenge, three mice per group were sacrificed and their lungs, spleens, livers and blood were collected individually. The tissue homogenates (in 800 uL of sterile PBS) and whole blood were serially diluted and plated on tryptic soy agar (TSA) plates, followed by incubation at 37°C for 8 h. Bacterial colonies were enumerated, and the corresponding concentration (CFU/g or CFU/mL) calculated.

### Histopathology

At both 21 days after the third immunization and 2 days post-challenge, part of the lung, liver, and spleen of mice were collected. Each organ was immediately placed in 4% paraformaldehyde for at least 24 h prior to being processed. Sectioned tissues were stained with hematoxylin and eosin (HE) prior to evaluation. Pathological alterations in tissue slices were observed by light microscopy. Tissue sections were evaluated by a trained pathologist, blinded to treatment and according to the following scores: 0, no pathological lesions; 1, minimal; 2, mild; 3, moderate; 4, severe.

### Statistics

Data are expressed as mean ± SD. All statistical analyses were performed using GraphPad Prism. Differences in the levels of antibodies among all groups of mice were assayed by two-way analysis of variance (ANOVA), followed by least significant difference (LSD) analysis or Tukey’s test. Survival rate was analyzed using Kaplan–Meier survival estimates. Comparisons were considered significantly different if *P <*0.05.

## Results

### Characterization of Inhalable rPA Vaccines

**Figure 2:**
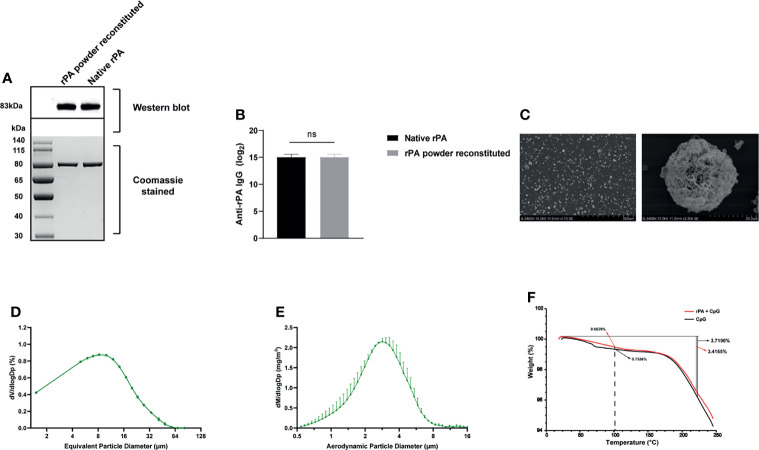
Characterization of rPA dry powder. **(A)** Western blot analysis of rPA dry powder and rPA liquid using mouse anti-rPA polyclonal antibodies (upper panel). A protein gel stained with Coomassie brilliant blue was used as a loading control (lower panel). **(B)** The immunogenicity of rPA dry powder and rPA liquid was analyzed by ELISA using mouse anti-rPA polyclonal antibodies. **(C)** Scanning electron microscopy images of rPA dry powder. **(D)** VMD of the aerosolized rPA dry powder, as determined using a laser particle size analyzer. **(E)** MMAD of the aerosolized rPA dry powder, as measured using the APS spectrometer 3321. **(F)** TGA of the rPA dry powder and CpG dry power (control). ns, not significant. rPA, recombinant protective antigen; ELISA, enzyme-linked immunosorbent assay; VMD, volume median diameter; MMAD, mass median aerodynamic diameter; APS, aerodynamic particle sizer; TGA, thermogravimetric analysis.

Three different formulations of rPA vaccines for i.t. immunization were prepared, including liquid, dry powder, and powder reconstituted in PBS. To check for any changes in rPA integrity that might occur due to the SFD process, powder samples were reconstituted for SDS–PAGE analysis (**Figure 2A**). The molecular weight of reconstituted rPA powder was identical to that of the liquid formulation (~83 kDa), indicating that the SFD process did not affect the integrity of rPA. Both reconstituted powder and liquid formulations reacted with mouse polyclonal antibodies to rPA, and titers did not differ significantly (**Figures 2A, B**), demonstrating that the immunogenicity of rPA was not affected by the SFD process.

Particle morphology was evaluated by scanning electron microscope. As shown in **Figure 2C**, the SFD powder were spherical and very porous, without any evident collapse or obvious shrinkage. The VMD of the rPA powder was 10.03 µm as measured by a laser particle size analyzer (**Figure 2D**). The MMAD of rPA aerosol particles, as measured by an APS spectrometer 3321, was 2.76 ± 0.06 µm (**Figure 2E**). Moisture content of the rPA powder was 0.663% w/w, as determined by TGA (**Figure 2F**). These results indicated that the rPA vaccine powder prepared was suitable for aerosol inhalation.

### Humoral Immune Response of Mice Immunized With rPA Vaccines

Prior to assessment of the vaccines, a B10.D2-Hc^0^ mice model of *B. anthracis* i.t. infection was established. The LD_50_ of i.t. *B. anthracis* Pasteur II spore challenge was 2.5×10^3^ CFU, which is about 100 times lower than that in C57BL/6J mice (3×10^5^ CFU, **Table S2** and **Figure S1**). Given this susceptibility of the B10.D2-Hc^0^ mouse to the *B. anthracis* attenuated strain, it was selected in subsequent immunization and challenge experiments to assess rPA vaccines with different formulations and immunization routes.

**Figure 3:**
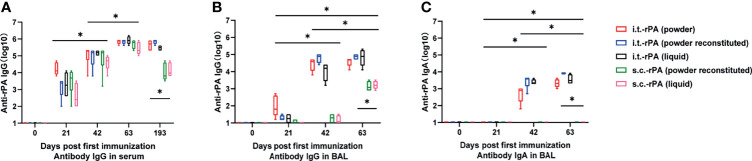
Humoral and mucosal immune responses after each vaccination in mice from each of the following 5 groups: (1) i.t.-rPA (powder), (2) i.t.-rPA (powder reconstituted), (3) i.t.-rPA (liquid), (4) s.c.-rPA (powder reconstituted), or (5) s.c.-rPA (liquid). **(A)** The reciprocal titers of IgG to rPA in mice serum. **(B)** The reciprocal titers of IgG to rPA in mice BAL. **(C)** The reciprocal titers of IgA to rPA in mice BAL. Four serum samples were collected at 0, 21, 42, 63 and 193 dppi and four BAL samples were collected at 0, 21, 42 and 63 dppi per group. Statistical differences were calculated by two-way ANOVA, followed by least significant difference (LSD) analysis or Tukey’s test. *P < 0.05. i.t., aerosolized intratracheal inoculation; s.c., subcutaneous injection; ANOVA, analysis of v ariance; LSD, least significant difference.

To evaluate the humoral immune response in mice immunized with the three rPA vaccine formulations (powder, powder reconstituted and liquid) *via* different routes (i.t. and s.c.), serum levels of rPA-specific IgG following immunization were measured. A substantial and progressive induction of anti-rPA antibody was observed in all groups (**Figure 3A**). Booster immunizations with the same formulation used in the primary vaccine significantly increased anti-rPA antibody levels at 42 and 63 dppi. After every immunization, rPA vaccines administered *via* i.t. elicited similar levels of serum anti-rPA IgG as those administered *via* s.c. (*P >*0.05). In addition, responses to different vaccine formulations did not differ (*P >*0.05). To assess the longevity of the antibody response to immunization, blood samples of mice were collected at approximately 6 months post-primary immunization; anti-rPA antibody titers in i.t. groups were maintained at 1:100000 over the course of 193 dppi, while titers in s.c. groups decreased to 1:1000, indicating that i.t. immunization with rPA induced a long-lasting anti-PA IgG antibody response that persisted for at least 193 dppi. As expected, no anti-rPA antibodies were detected in any PBS- and CpG-immunized mice (data not shown).

### Lung Mucosal Immune Response of Mice Immunized With rPA Vaccines

To investigate whether i.t. inoculation improves rPA-induced mucosal immunity, antigen-specific IgG and SIgA were analyzed in BAL collected at 0, 21, 42 and 63 dppi. IgG production increased in i.t. groups after the booster immunizations (days 42 and 63; **Figure 3B**). Vaccination *via* s.c. induced low titers of anti-rPA mucosal IgG even after the third immunization. Different vaccine formulations induced comparable anti-rPA IgG titers at all time points in both i.t. and s.c. groups.

Similar trends were seen in anti-rPA SIgA titers (**Figure 3C**), with the notable exception that vaccines delivered *via* s.c. did not induce specific SIgA in BAL at any of the time points tested. All i.t. groups including powder, powder reconstituted, and liquid groups showed a continuous immune response with moderate increase in anti-rPA SIgA titers, which reached 1:1000 at 63 dppi. Thus, i.t. inoculation of rPA vaccines apparently induces lung mucosal immune response.

### Aerosolized Intratracheal Inoculation of rPA Induced High Titers of Neutralizing Antibodies Against Anthrax Toxin in Mice

**Figure 4:**
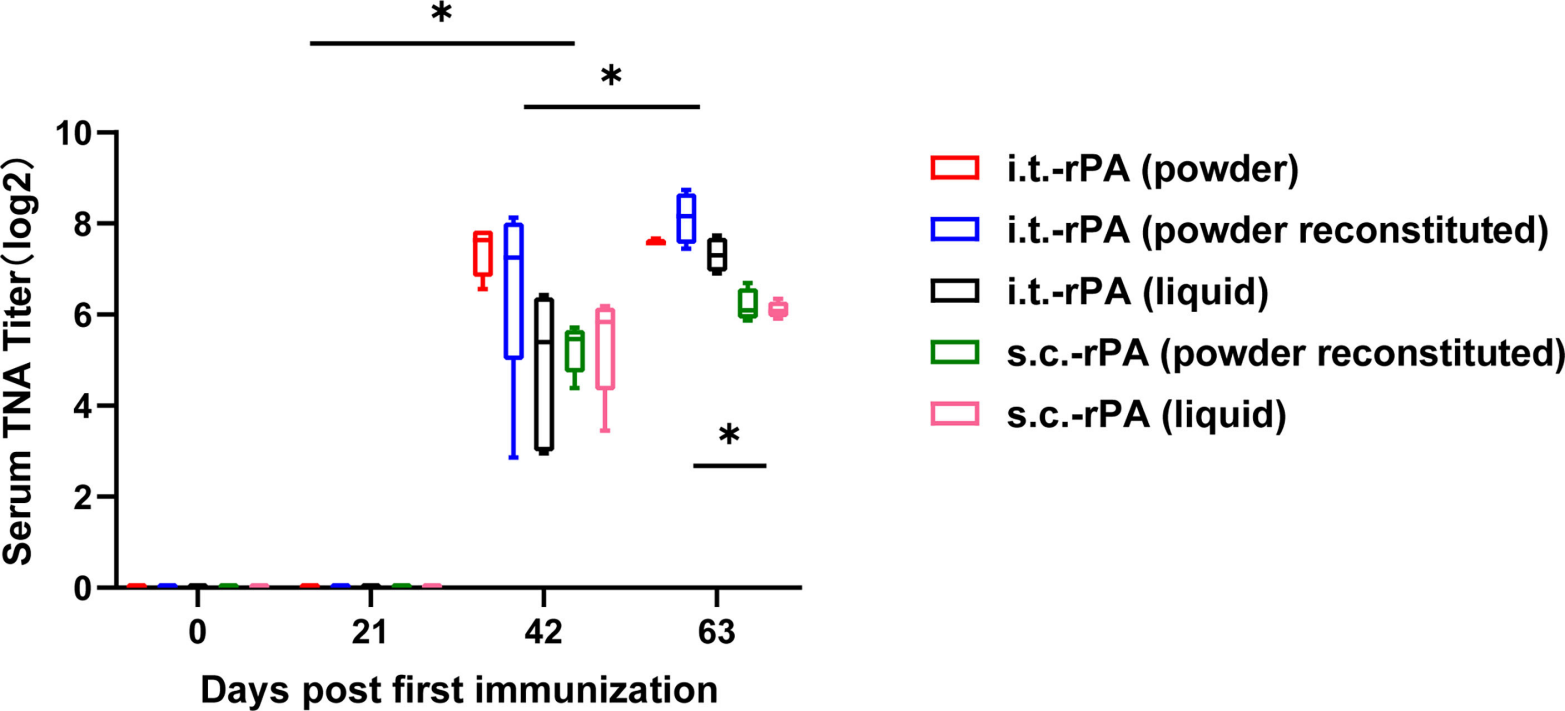
The reciprocal titers of LeTx neutralizing antibodies to rPA in mice serum. Titer values were determined as the inflection point of the antibody dilution curve reported as the effective dilution at 50% inhibition and were log_2_ transformed. Statistical differences were calculated by two-way ANOVA, followed by LSD analysis or Tukey’s test. *P < 0.05. LeTx, lethal toxin.

Serum collected from immunized mice at 0, 21, 42 and 63 dppi was tested for ability to neutralize anthrax. The serum dilution required to neutralize 50% of the LeTx (LeTx-ED_50_) was measured. A detectable antibody response was observed at 42 dppi in all groups, although responses were highly variable within groups (**Figure 4**). The LeTx-ED_50_ titers had higher values and less within-group variability at 3 weeks after the last immunization (63 dppi). At this time point, serum from mice immunized *via* i.t. were more effective in neutralizing LeTx and preventing J774A.1 cells’ death compared with those from s.c. groups (**Figure 4**). No significant differences in the LeTx-ED_50_ titers were present between the three i.t.-rPA-immunized groups (liquid, powder, powder reconstituted).

### Cellular Immune Response Induced by Aerosolized Intratracheal Inoculation of rPA

**Figure 5:**
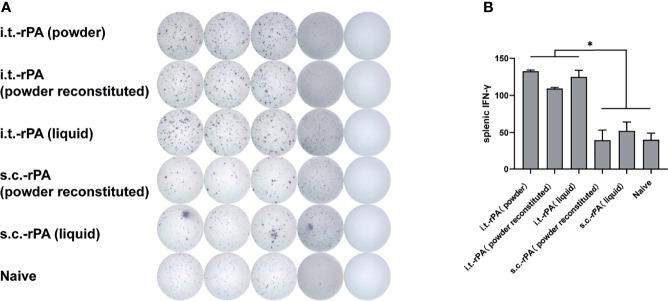
IFN-γ ELISPOT-based quantification of antigen-specific T cells in mice. At 63 dppi, T cells were isolated from the spleens of three mice in each group and stimulated with rPA for 40 h **(A)** elispot result measured by Biosys Bioreader 7000. **(B)** quantification of antigen-specific IFN-γ-producing T cells. Data are expressed as mean ± SD (n = 3). *P < 0.05. IFN-γ, interferon-γ; ELISPOT, enzyme-linked immunospot. SD, standard deviation.

To further understand the T cell immune response elicited by different rPA formulations and immunization routes, IFN-γ and IL-4 levels in splenic cells isolated from immunized mice were evaluated using ELISPOT analysis. Secretion levels of IFN-γ in i.t.-rPA-immunized groups were significantly higher than those of s.c.-rPA-immunized groups and control groups (*P <*0.05; **Figures 5A, B**), indicating that i.t.-immunization was more effective than s.c.-injection in inducing cellular immune response. However, no significant differences in IL-4 secretion were observed between rPA-immunized groups and control groups (data not shown).

### Protection of Mice From i.t. Challenge With *B. anthracis* Spores

**Figure 6:**
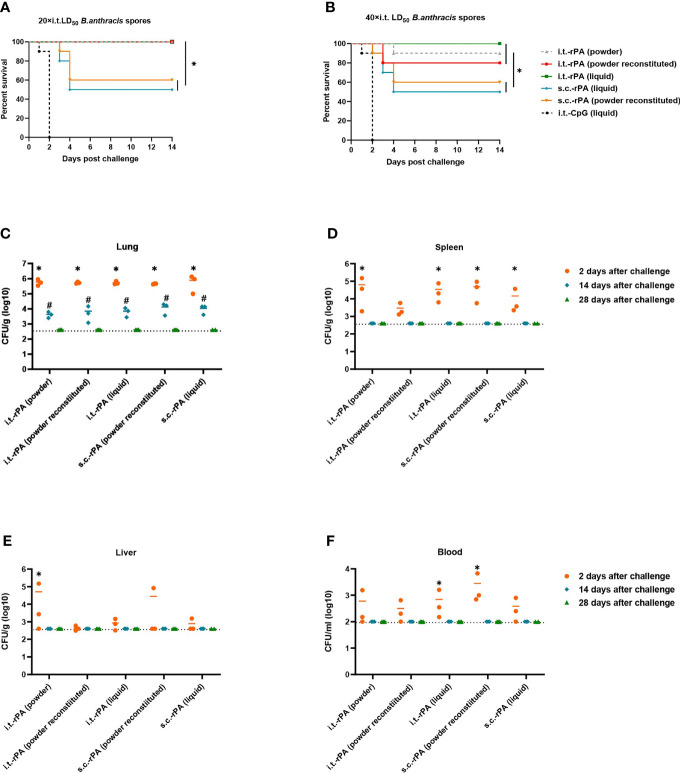
Protection of the rPA vaccines in mice against *B*. *anthracis* challenges. Mice were immunized (i.t. or s.c.) three times with rPA vaccines according to **Figure 1A** and challenged at day 21 after the third vaccination. **(A)** Survival of mice (n = 10) against 20× LD_50_ of aerosolized *B*. *anthracis* Pasteur II spores. **(B)** Survival of mice (n = 10) against 40× LD_50_ aerosolized *B*. *anthracis* Pasteur II spores. **(C–F)** Bacterial and spore loads of mice euthanized at days 2, 14, and 28 post 40× LD_50_ of aerosolized *B*. *anthracis* Pasteur II spores challenge in **(C)** lungs, **(D)** spleens, **(E)** livers and **(F)** blood. The limits of detection were 400 CFU for the lungs, spleen and liver, and 100 CFU for blood. *P < 0.05 in **(A, B)**; *P < 0.05 compared to 14 days after challenge, ^#^ *P <* 0.05 compared to 28 days after challenge in **(C–F)**. Challenge experiments with 40× LD_50_ Pasteur II spores were performed twice in different batches immunized mice.

The protective efficacy of i.t. inoculation with different rPA vaccine formulations against 20× LD_50_ or 40× LD_50_ of i.t. *B. anthracis* Pasteur II spore challenge was evaluated in B10.D2-Hc^0^ mice model. All animals in the PBS and CpG groups succumbed to infection within 2 d post-spore challenge. For clarity, only the i.t.-CpG (liquid)-immunized group is shown in the figures. At the lower challenge level of 20× LD_50_, mice vaccinated with any of the three formulations of rPA *via* i.t. had 100% protection (**Figure 6A**). In s.c.-immunized groups, rPA in liquid and powder reconstituted formulations yielded survival rates of 50% and 60%, respectively, which were significantly lower than those of i.t.-rPA groups. However, survival between the two vaccine formulations did not differ significantly.

At the higher challenge level of 40× LD_50_, i.t. immunization again provided greater protection than s.c. immunization (**Figure 6B**). Mice in the i.t.-rPA (liquid)-immunized group were fully protected. One of 10 mice died in the i.t.-rPA (powder)-immunized group and two of 10 died in the i.t.-rPA (powder reconstituted)-immunized group, and no statistically significant difference was present among the three i.t. groups. The corresponding vaccines delivered *via* s.c. protected no more than 60% of the mice. Challenge experiments were performed twice.

Bacterial and spore loads of tissues (lung, spleen, liver and blood) in mice receiving 1×10^5^ CFU spores (40× LD_50_) are shown in **Figures 6C–F**. As no PBS- or CpG-group mice survived to day 2 post-challenge, data from these groups were not available. For lungs, all mice had bacterial and spore loads ranging from 10^5^ to 10^6^ at day 2 post-challenge that decreased to 10^3^ at day 14 post-challenge. No bacteria were detected in the lowest dilution at day 28 post-challenge, indicating clearance of the bacteria. For spleen, liver and blood, detectable bacteria were present in all five groups at day 2 post-challenge, except for liver, where one to two (out of three) mice had undetectable bacteria in the i.t.-rPA (powder)-, s.c.-rPA (powder reconstituted)- and s.c.-rPA (liquid)-immunized groups. Unlike lungs, bacteria in spleen, liver and blood were cleared by day 14 post-challenge.

### Pathological Alterations in Mouse Organs After Vaccination and Challenge

No obvious pathological lesions were observed in the lungs, spleens or livers of mice immunized with any formulation of rPA, CpG, or PBS *via* i.t (**Figure S2A**), confirming the safety of i.t.-rPA immunization.

Pathological alterations were also examined in mice receiving 1×10^5^ CFU spores (40× LD_50_). Tissues were collected from rPA-immunized mice at day 2 post-challenge and were collected from naive-infected mice that were moribund, regardless of the day post-challenge. In naive-infected mice, neutrophil infiltration, hemorrhage and consolidation were observed in lungs and multifocal lymphocytic infiltration was observed in livers (**Figure S2B**). Necrotizing splenitis and numerous bacterial colonies were observed in spleens. In rPA-immunized mice, there were only mild inflammatory responses in lungs and livers. The pathological scores of lungs, spleens and livers in rPA-immunized groups were significantly lower than that of naive-infected control groups (**Figures S2C–F**).

## Discussion

*B. anthracis* has become a biological warfare agent of major concern because it is capable of infecting hundreds of thousands of individuals with a single aerosol dispersion (39), and this has spurred increased interest in *B. anthracis* and in efforts to improve vaccines and treatments against anthrax. In this study, we prepared anthrax vaccine in powders containing rPA with the adjuvant CpG using a controlled SFD condition, to create particle physicochemical properties appropriate for i.t. inoculation. The potency and efficacy of three rPA formulations delivered *via* two immunization routes against *B. anthracis* Pasteur II strain spore infection were assessed in B10.D2-Hc^0^ mice, which are deficient in complement component C5. Studies have shown that mice lacking complement component C5 are susceptible to attenuated Sterne anthrax infection (28). Pasteur II is the attenuated anthrax vaccine strain used for the immunization of livestock. It was thought to have lost the pXO1 plasmid due to exposure to high temperatures during subculture (40) but a recent study indicates the presence of a low copy number of pXO1 plasmid DNA using more sensitive methods, signifying that it can produce toxins (41). The attenuation of this vaccine strain is likely due to the impact of high temperature stress on plasmid replication, which in turn limits the copy number of pXO1. Thus, it may better mimic the natural infection compared with the Sterne strain with no pXO2, while still avoiding the need to use of virulent strains in high-level biosafety laboratories.

Three significant insights stem from this study. First, i.t. inoculation of rPA vaccines enhances protection efficacy against inhalational anthrax in mice. In clinical trials, rPA is currently administered *via* i.m. or s.c. using conventional needles and syringes, but no data exists to indicate these routes are optimal. Rabbits immunized with rPA *via* i.m. injection had 70% protection from an aerosol spore challenge (42). A similar experiment with guinea pigs found partial protection with the same route (43). In our study, i.t. inoculation of rPA vaccine elicited 100% protection against a 20× LD_50_ Pasteur II spore challenge regardless of vaccine formulation, compared with only 50% for s.c. injection with liquid rPA.

Second, i.t. inoculation of rPA vaccines induces a higher LeTx neutralizing antibody titer, a stronger lung mucosal immune response and a greater cellular immune response than s.c. injection. One of the great advantages of mucosal vaccines is the possibility to induce not only serum antibodies but also a mucosal immune response at the local entry point of pathogens (44). In the current study, a continuous systemic immune response was observed among all groups after the third immunization, with an anti-rPA IgG titer of 10^5^ in serum. No significant difference occurred between i.t. and s.c. immunization. However, in LeTx neutralizing antibody titers, mice immunized *via* i.t. were more effective in neutralizing LeTx compared to s.c. groups. These results agree with other studies that have shown no precise correlation between antibody titer to PA and protection against challenge exists in mice and guinea pigs (45, 46), but that a positive correlation between LeTx neutralizing antibody titers and survival does (47).

Various studies have confirmed the importance of mucosal immunity in protection against pathogens that enter the body through the mucosal surface. Secretory IgA (SIgA) is the predominant immunoglobulin at the mucosal surfaces. Previous studies showed that IgA antibodies are necessary for the development of a protective immune response to rotavirus (48) and are superior to IgG in protecting primates from a mucosal challenge with simian-human immunodeficiency virus (SHIV) (49). However, the role of mucosal immunity in protective efficacy against inhalational anthrax remains unclear. In this study, i.t. inoculation of rPA led to a significant concentration of mucosal anti-rPA SIgA, whereas none of the mice from s.c. groups had a detectable SIgA titer in BAL. Interestingly, a previous study showed no detectable mucosal response was induced with an intranasal vaccine consisting of rPA and CpG (50), which is also a mucosal vaccination. This discrepancy is likely due to the different delivery routes, indicating that i.t. inoculation may have more potential to elicit mucosal immunity, compared with. intranasal delivery. Moreover, a weak LeTx neutralization activity was induced in the BAL of i.t.-immunized mice, but not s.c.-immunized mice, as expected (**Figure S3**). Together, these results provide further explanation for the better protection observed in i.t.-immunized groups.

The third important insight from our study, is that powder formulation immunization *via* i.t. may be a potent alternative that improves on the existing vaccination. For the three formulations (liquid, powder and reconstituted powder) we tested, no significant differences existed between serum anti-rPA IgG and BAL anti-rPA SIgA titers in either the i.t. or s.c. groups. Similar results were observed for survival rates following i.t. or s.c. vaccinations. It is notable that in our high-dose *B. anthracis* spores of the i.t.-challenge experiment, these three rPA formulations using i.t. delivery route conferred protection of 100%, 90% and 80%, respectively, although no statistically significant difference was found. Additional experiments are needed to confirm whether survivorship varies among formulations. However, a recent study showed that vaccine in liquid formulation provided a slightly lower protection than powder (51), which may because of the different animal model. Overall, under the premise of comparable potency and efficacy, powder vaccine formulation has advantages over liquid formulation; it offers the potential to eliminate preservatives and the cold-chain requirement for shipping and storage. Dry powder vaccine with i.t. delivery may provide the optimal approach for developing a stable and effective alternative to improve on current available anthrax vaccines.

In summary, our study indicates that i.t. immunization with rPA provided nearly complete protection against inhalational anthrax and induced a greater humoral and cellular response compared with s.c. immunization. Powder formulation provides a promising alternative to the existing vaccination. In addition, B10.D2-Hc^0^ mice with a *B. anthracis* Pasteur II challenge is a useful small animal model for anthrax vaccine candidate screening.

## Data Availability Statement

The original contributions presented in the study are included in the article/**Supplementary Material**. Further inquiries can be directed to the corresponding authors.

## Ethics Statement

The animal study was reviewed and approved by Beijing Institute of Microbiology and Epidemiology.

## Author Contributions

Thanks to all authors for their contributions to this article. DZ, XX, and WY conceived and designed the study. XS, WZ, LZ, and LLZ performed animal experiments. JG, YZ, LH, and ML performed other experiments. XS and WZ contributed to data analysis and interpretation and writing the first draft of the manuscript. DZ, XX, WY, and ML revised the manuscript. All authors contributed to the article and approved the submitted version.

## Conflict of Interest

The authors declare that the research was conducted in the absence of any commercial or financial relationships that could be construed as a potential conflict of interest.‬

## Publisher’s Note

All claims expressed in this article are solely those of the authors and do not necessarily represent those of their affiliated organizations, or those of the publisher, the editors and the reviewers. Any product that may be evaluated in this article, or claim that may be made by its manufacturer, is not guaranteed or endorsed by the publisher.
