# Supplementary material for: Aerosolized Intratracheal Inoculation of Recombinant Protective Antigen (rPA) Vaccine Provides Protection Against Inhalational Anthrax in B10.D2-Hc0 Mice
Source: Front Immunol. 2022 Jan 26;13:819089. doi: 10.3389/fimmu.2022.819089 (PMC8826967; doi:10.3389/fimmu.2022.819089)
Supplement: Supplementary file 1 [file DataSheet_1.pdf]

Supplementary Material

1 Supplementary Figures and Tables

1.1 Supplementary Tables

SUPPLEMENTARY TABLE 1 Composition and proportion of rPA spray-drying solution

Composition	Proportion	Properties
rPA	0.1% (w/v)	Antigen
CpG	0.1% (w/v)	Adjuvant
D-mannitol	1%(w/v)	Increases dispersibility
Myo-inositol	1% (w/v)	Enhances stability
L-leucine	0.3% (w/v)	Enhances dispersibility and stability
Poloxamer 188	0.05% (v/v)	Enhances stability, surfactants

SUPPLEMENTARY TABLE 2 Survival rates of B10.D2-Hc^{0} and C57BL/6J mice challenged with different doses of *B. anthracis* Pasteur II spores

Challenge route	B10.D2-Hc^{0}		C57BL/6J	
	Challenge dose (CFU)	Survivors/no. challenged (%)	Challenge dose (CFU)	Survivors/no. challenged (%)
i.t.[a]	5×10^{2}	8/10 (80)	1×10^{3}	10/10 (100)
	1×10^{3}	6/10 (60)	1×10^{4}	9/10 (90)
	2.5×10^{3}	4/10 (40)	1×10^{5}	7/10 (70)
	5×10^{3}	0/10 (0)	1×10^{6}	2/10 (20)
	1×10^{4}	0/10 (0)	1×10^{7}	0/10 (0)

[a] i.t., aerosolized intratracheal inoculation.

1.2 Supplementary Figures

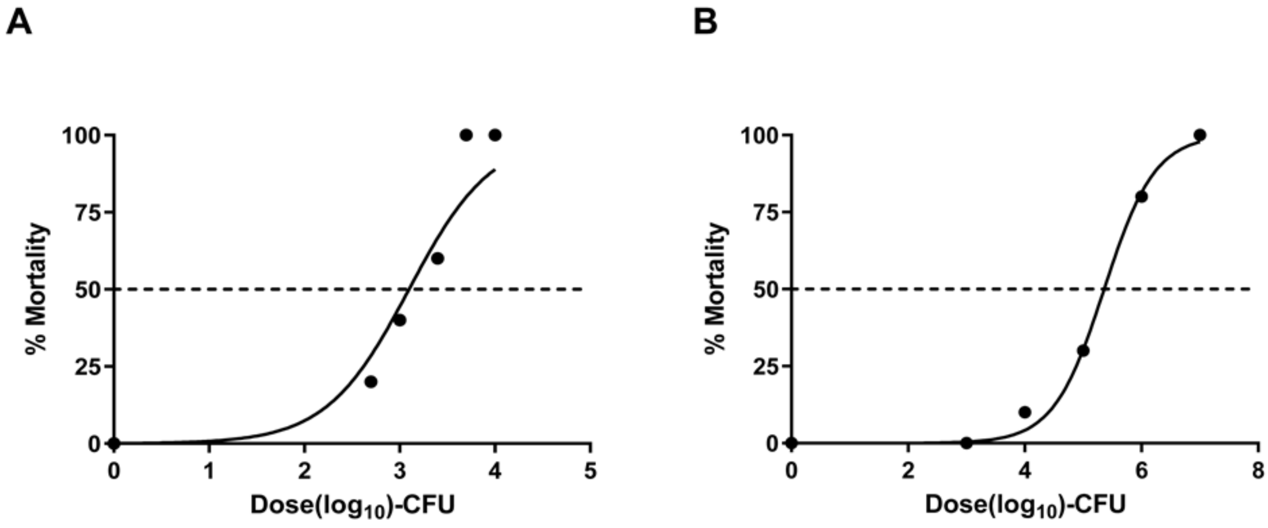

SUPPLEMENTARY FIGURE 1. Lethality of mice challenged with i.t. *B. anthracis* Pasteur II spores. **(A)** B10.D2-Hc^{0} mice. **(B)** C57BL/6J mice.

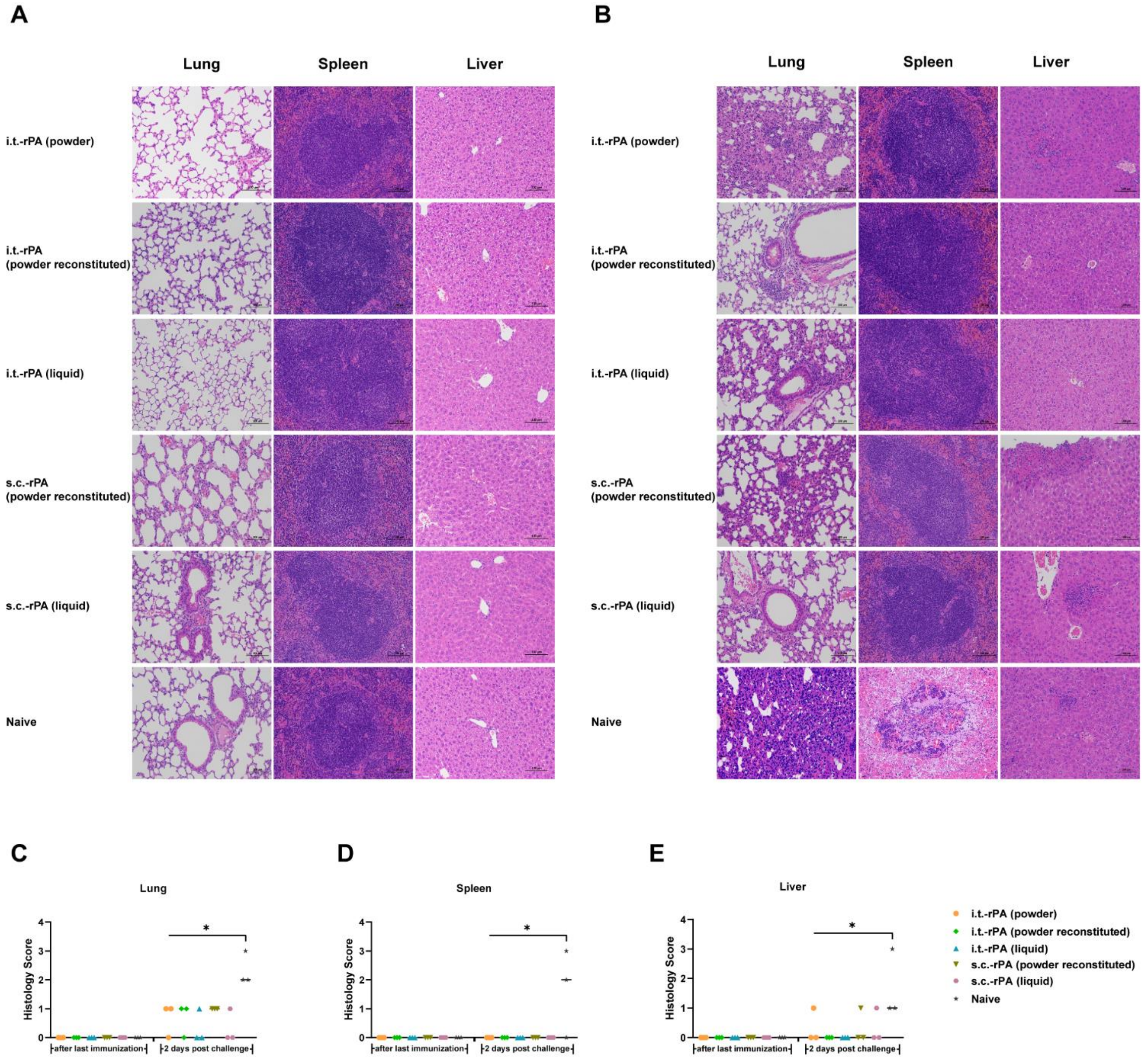

SUPPLEMENTARY FIGURE 2. Pathological changes in the tissues of mice after immunization and challenge. Three mice per group were euthanized 21 days after the third immunization and day 2 post 40×LD_{50} i.t. challenge. Part of their lungs, spleen and liver were collected. The tissues collected **(A)** after the third immunization and **(B)** after i.t. challenge were tested using conventional histopathological methods and observed in HE stained sections by light microscopy. Tissue sections of **(C)** lungs, **(D)** spleens and **(E)** livers were evaluated by a trained pathologist according to the following scores: 0, no pathological lesions; 1, minimal; 2, mild; 3, moderate; 4, severe.

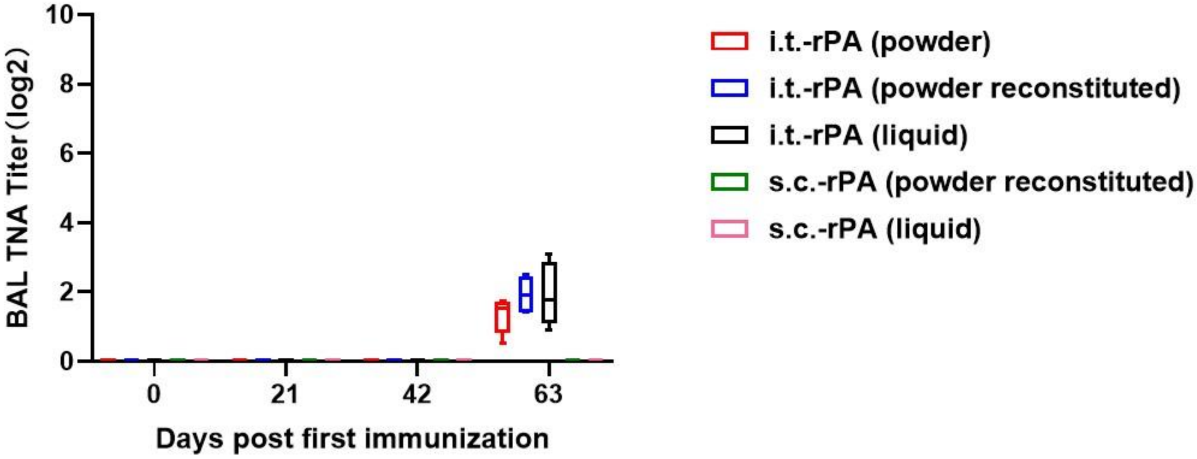

SUPPLEMENTARY FIGURE 3. The reciprocal titers of LeTx neutralizing antibodies to rPA in mice BAL. Titer values were determined as the inflection point of the antibody dilution curve, reported as the effective dilution at 50% inhibition (ED_{50}) and were log2 transformed. No detectable LeTx neutralizing antibody was observed in all s.c.-immunized mice BAL. Weak LeTx neutralization activities were observed in i.t.-immunized mice BAL after the third immunization.
